# Molecular characterization of SQUAMOSA PROMOTER BINDING PROTEIN-LIKE (SPL) gene family from *Citrus* and the effect of fruit load on their expression

**DOI:** 10.3389/fpls.2015.00389

**Published:** 2015-05-27

**Authors:** Liron Shalom, Lyudmila Shlizerman, Naftali Zur, Adi Doron-Faigenboim, Eduardo Blumwald, Avi Sadka

**Affiliations:** ^1^Department of Fruit Tree Sciences, Agricultural Research Organization, The Volcani CenterBet Dagan, Israel; ^2^The Robert H. Smith Faculty of Agriculture, Food and Environment, The Hebrew University of JerusalemRehovot, Israel; ^3^Department of Plant Sciences, University of California, DavisDavis, CA, USA

**Keywords:** alternate bearing, *Citrus*, flowering, *miR156*, *miR172*, *SPL*

## Abstract

We recently identified a *Citrus* gene encoding SQUAMOSA PROMOTER BINDING PROTEIN-LIKE (SPL) transcription factor that contained a sequence complementary to *miR156*. Genes of the SPL family are known to play a role in flowering regulation and phase transition. In *Citrus*, the mRNA levels of the gene were significantly altered by fruit load in buds; under heavy fruit load (ON-Crop trees), known to suppress next year flowering, the mRNA levels were down-regulated, while fruit removal (de-fruiting), inducing next-year flowering, resulted in its up-regulation. In the current work, we set on to study the function of the gene. We showed that the *Citrus* SPL was able promote flowering independently of photoperiod in *Arabidopsis*, while *miR156* repressed its flowering-promoting activity. In order to find out if fruit load affected the expression of additional genes of the SPL family, we identified and classified all SPL members in the *Citrus* genome, and studied their seasonal expression patterns in buds and leaves, and in response to de-fruiting. Results showed that two additional *SPL*-like genes and *miR172*, known to be induced by SPLs in *Arabidopsis*, were altered by fruit load. The relationships between these factors in relation to the fruit-load effect on *Citrus* flowering are discussed.

## Introduction

During their growth, plants undergo a series of developmental transitions, regulated by a complex network of molecular factors that are activated and interact in response to endogenous and environmental cues. Research of juvenile-to-adult and vegetative-to-reproductive phase transitions in model and crop plants has revealed the importance of two microRNAs, *miR156* and *miR172*, which coordinate these processes in an opposite manner: *miR156* is highly abundant during the juvenile phase and gradually decreases with age; its overexpression prolongs the expression of juvenile features and significantly delays flowering (Wu and Poethig, 2006; Chuck et al., 2007); in contrast, *miR172* abundance increases as the plant ages and its overexpression accelerates flowering (Aukerman and Sakai, 2003; Chen, 2004; Lauter et al., 2005; Wu and Poethig, 2006; Jung et al., 2007). In *Arabidopsis*, *miR156* targets 10 out of 16 members of the SQUAMOSA PROMOTER BINDING PROTEIN-LIKE (SPL) family of transcription factors (TFs), which are characterized by a 76-amino acid DNA-binding domain named SBP. SPLs influence flowering in a number of ways. In the leaf, they promote flowering upstream of FLOWERING LOCUS T (FT) by up-regulating *miR172*, a repressor of APETALA2 (AP2)-like TFs which inhibits *FT* transcription (Mathieu et al., 2009). In the apex, the expression of several *SPLs* increases during early stages of flowering transition (Wang et al., 2009) and further activates important MADS-box genes by directly binding their promoters (Wang et al., 2009; Yamaguchi et al., 2009). In addition, flowering via the gibberellin (GA) pathway is mediated by SPLs (Yu et al., 2012), whereas the *miR156*–*SPL* module directly regulates *FT* expression to control ambient temperature-responsive flowering (Kim et al., 2012). SPLs have also been identified as potential targets of the signaling molecule trehalose-6-phosphate, which positively regulates flowering in *Arabidopsis* (Wahl et al., 2013), suggesting a relationship between SPLs and the carbohydrate status of the plant. Aside from their regulatory role in floral transition, SPLs have been found to influence diverse physiological processes, such as sporogenesis (Unte et al., 2003), leaf development (Usami et al., 2009), copper homeostasis (Yamasaki et al., 2009), male fertility (Xing et al., 2010), gynoecium patterning (Xing et al., 2013), trichome development (Yu et al., 2010) and anthocyanin biosynthesis (Gou et al., 2011). The involvement of *miR156*, *miR172*, and SPLs in the juvenile-to-adult phase transition has also been demonstrated in woody perennials, including English ivy, eucalyptus, poplar, acacia and oak, in a manner similar to that in annual plants (Wang et al., 2011).

In addition to the age-dependent phase transition, perennials undergo seasonal phase transitions into flowering, which usually occur on an annual basis. However, in some tree species, flowering occurs biennially or even every few years. In biennial bearers, heavy fruit load 1 year (ON-Crop year) inhibits flowering the following year (OFF-Crop year), a phenomenon known as alternate bearing (AB) (Monselise and Goldschmidt, 1982). Complete fruit removal (de-fruiting) from ON-Crop trees induces next-year flowering (return bloom). In a search for regulatory and other processes which are altered in the buds as a result of contrasting fruit loads in *Citrus*, we previously identified an *SPL*-like gene whose mRNA level was relatively high during OFF-Crop years and low during ON-Crop years (Shalom et al., 2012). Moreover, de-fruiting significantly induced this gene's expression in the bud within a short time (Shalom et al., 2014). Although this *SPL*-like gene contained a *miR156*-binding site, no changes in *miR156* levels were detected between buds of ON- and OFF-Crop trees, suggesting its regulation by other factors as well.

To the best of our knowledge, the above studies were the first to demonstrate a correlation between *SPL*-like gene expression and fruit load in fruit trees. However, further analyses are required to strength these relationships. In the current work, we performed a functional analysis of the *Citrus SPL* in *Arabidopsis*. The results showed it was able to promote flowering, while *miR156* repressed its action. As in *Arabidopsis*, the *Citrus* genome contains additional *SPL*-like genes. Therefore, we also set out to identify all *SPL*s from *Citrus* and characterize their expression throughout the season and following de-fruiting. The responses of *miR156* and *miR172* to de-fruiting in *Citrus* buds are also characterized, and the relationships between them and *SPL*s with respect to AB are discussed.

## Materials and methods

### Identification and phylogeny of *Citrus* SPLs

*Citrus SPL* genes were identified using the *Citrus clementina* genome (http://www.phytozome.net). *Arabidopsis SPL* sequences were compiled from the TAIR database (https://www.arabidopsis.org/). Alignments were performed by MUSCLE program using default parameters (http://www.ebi.ac.uk/Tools/msa/muscle/ (Edgar, 2004). A phylogenetic tree was constructed based on the maximum likelihood (ML) framework using Phyml software (Guindon and Gascuel, 2003) by the JTT matrix-based model. The tree was graphically designed with the use of FigTree version 1.4 (http://tree.bio.ed.ac.uk/software/figtree/).

### Full-length sequencing of *CiSPL5* mRNA

An Expressed Sequence Tags (EST) based *SPL* consensus sequence was pooled from the HarvEST *Citrus* database (http://harvest.ucr.edu/) according to the probe set ID of the Citrus GeneChip Microarray (Affymetrix, Inc., Santa Clara, CA). Total RNA was extracted from buds of 15-year-old Murcott mandarin (*Citrus reticulata* Blanco) trees grafted on sour orange (*Citrus aurantium* L.), using the CTAB extraction method (Chang et al., 1993). RNA was treated with RQ1 RNase-free DNase (Promega, Fitchburg, WI) according to the manufacturer's instructions. 5′-RACE and 3′-RACE for *CiSPL5* were carried out using the FirstChoice RLM RACE Kit (Ambion, Austin, TX). For 5′-RACE, 10 μg of total RNA was ligated to the RNA adapter after treatment with calf intestinal phosphatase and tobacco acid pyrophosphatase, followed by cDNA synthesis using random primers. For 3′-RACE, cDNA was synthesized using the oligo d(T) adapter supplied by the manufacturer. Outer PCR and inner PCR were carried out using the adapter primers, and primers specific for *Citrus SPL* (Supplementary Table 4). RACE products were gel-purified and cloned into pGEM T easy vector (Promega) for sequencing.

### Plasmid construction and production of transgenic plants

The *CiSPL5* transcript sequence (open reading frame [ORF] + 3′ untranslated region [UTR]) and a sequence lacking the 3′UTR (ORF no 3′UTR) were PCR-amplified with Pfu DNA polymerase (Thermo Fisher Scientific, Waltham, MA) using cDNA as the template. *CiSPL5* ORF + 3′UTR was amplified using the following primers: forward CTAAAGGAAAAGACTGTCAAGGATT, reverse GCGTAACGATTGATTCCTCAG. *CiSPL5* ORF no 3′UTR was amplified using the following primers: forward CTAAAGGAAAAGACTGTCAAGGATT, reverse CTACTGAGGACCTACCCCTC. The sequence *CiSPL5* ORF + mutated 3′UTR was generated by introducing 10 mutations into the predicted *miR156*-binding site using recombinant PCR. Primers used for recombinant PCR were: forward TCGCATATTCACTACTCTCTTCCTTAGGCTCCTCCTCT, reverse AGAGAGTAGTGAATATGCGACCTGCAATGCAGAAAGTT in combination with the primers used for amplification of *CiSPL5* ORF + 3′UTR. All three constructs were cloned downstream of the CaMV 35S promoter in pART27 using pART7 as an intermediate vector (Gleave, 1992). *Arabidopsis* plants (Columbia ecotype) were transformed using the floral dip method (Bechtold et al., 1993). Transformed plants were identified by kanamycin selection (50 μg/ml). Plants were grown under long-day (16 h light) or short-day (8 h light) conditions at 22°C. Flowering time represents the appearance of the first open flower.

### *Citrus SPL*s expression analyses

Complete fruit removal (de-fruiting) and sample collection were carried out as previously described (Shalom et al., 2014). For seasonal expression analyses, plant material was collected from a commercial orchard of 15-year-old Murcott mandarin (*C. reticulata* Blanco) trees grafted on sour orange (*C. aurantium* L.), located in the central coastal area of Israel, during the year 2014. Samples were collected from three biological replicates, each containing three OFF-Crop trees. Vegetative shoots, collected from the southeast side of the trees, were taken to the laboratory on ice. Buds and leaves were separated and immediately frozen in liquid nitrogen. Total RNA was extracted using the CTAB extraction method (Chang et al., 1993) and treated with RQ1 RNase-free DNase (Promega) according to the manufacturer's instructions. Primers for all of the identified *Citrus SPL*s were designed based on genomic sequences (Phytozome, http://www.phytozome.net/) using Primer3 software (Supplementary Table 1). To exclude putative antisense transcripts (ASTs) as templates in the real-time PCR, primers were designed to span exon–exon junctions or two different exons with a large intron between them. Real-time PCR was carried out as described (Shalom et al., 2012).

### *miR156* and *miR172* analysis

*ctr-MIR156* and *csi-MIR172a* (one of several identified *miR172* genes) were previously identified as *Citrus* microRNA genes transcribing precursors which generate mature *miR156* and *miR172* sequences, respectively (Song et al., 2009; Xu et al., 2010). Thus, measuring their expression may indicate the abundances of their compatible mature miRNAs. In the two available *Citrus* genome databases (Phytozome and http://citrus.hzau.edu.cn/orange/), both *ctr-MIR156* and *csi-MIR172a* were not predicted as genes, probably duo to their relatively short lengths (<200 bp) and the lack of sufficient open reading frames, and are therefore represented here by their names given upon identification. For the analyses of *ctr-MIR156* and *csi-MIR172a* expression, *Citrus* microRNA (miRNA) precursor sequences (Supplementary Table 2) were compiled from the miRBase database (http://www.mirbase.org/). Abundance estimates were calculated for each sequence and sample using the RSEM software package (Li and Dewey, 2011) and Bowtie alignment program (Langmead et al., 2009). TMM (trimmed mean of *M*-values—weighted trimmed mean of the log expression values) normalization was performed using code in edgeR, as described by Robinson and Oshlack (2010), and applied to scale the FPKM-values provided by the abundance estimation software (RSEM) across all samples (Haas et al., 2013). The abundance of mature *miR156* and *miR172a* sequences was determined using TaqMan® Small RNA Assay Kits (Thermo Fisher scientific, Walthem, MA, USA) according to manufacturer's instructions; 10 ng total RNA was used, and real-time PCR was run in a Rotor Gene Q instrument (Qiagen, Venlo, Netherlands). The results were normalized against the *β-actin* gene as described previously (Shalom et al., 2014).

### Statistical analysis

Real-Time PCR results were analyzed by One-Way analysis of variance (ANOVA) with Tukey–Kramer multiple comparison tests, as implemented in the software JMP version 10 (SAS Institute, Cary, NC, USA).

## Results

### Gene structure and phylogeny of *SPL* family members from *Citrus*

**Figure 1:**
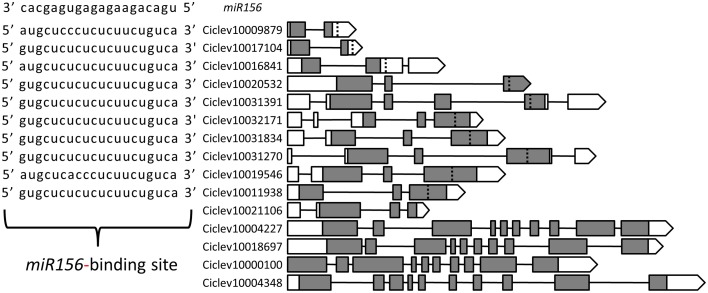
**Genomic structure of** ***Citrus SPL*** **genes.** Exon-intron organization of the primary transcript of each *SPL* gene is based on the *Citrus clementina* genome (http://www.phytozome.net/). Exons are represented by boxes and introns by connecting lines. White boxes represent un-translated regions and gray boxes represent coding regions. The dotted vertical lines indicate the location of the *miR156*-binding sites; their sequences are shown to the left.

**Figure 2:**
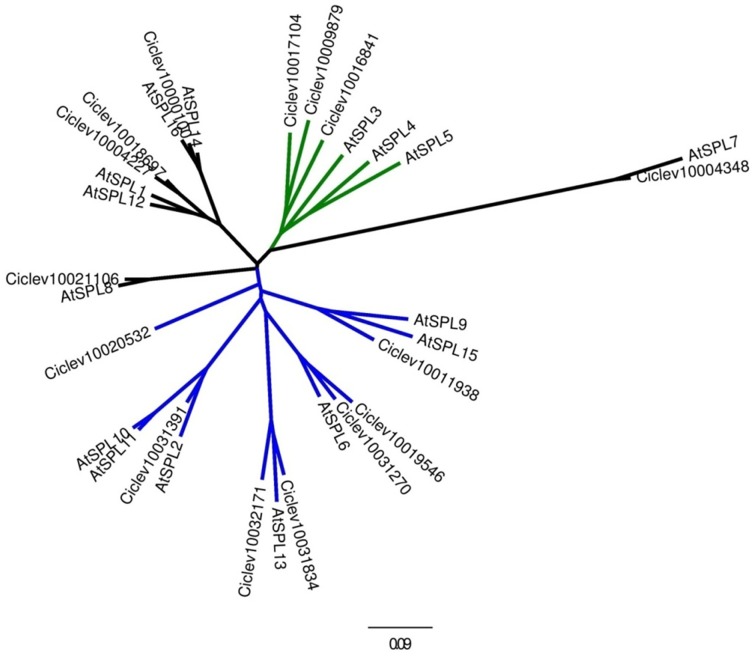
**Evolutionary relationships of SPL members from** ***Citrus*** **and** ***Arabidopsis***. The maximum likelihood tree is based on the SBP domains (Supplementary Table 3). SPLs were classified into three subgroups: <200 amino acids with *miR156*-binding site located within the 3′UTR (green), >300 amino acids with *miR156*-binding site located within the ORF (blue), >300 amino acids without *miR156* binding site (black).

To identify *Citrus SPL* members the following approaches have been taken: (1) all sequences containing an SBP domain were compiled from the *Citrus clementina* genome database (http://www.phytozome.net/), (2) proper identification of all matching sequences was confirmed by performing BLAST against two different *Citrus* genome databases (Phytozome and http://citrus.hzau.edu.cn/orange/) using the 16 SBP-domain sequences from *Arabidopsis* as queries, and (3) all *SPL*-related unigenes were pooled from the *Citrus* EST database (http://harvest.ucr.edu/) and BLASTed against the *C. clementina* genome database. Following removal of redundant sequences and alternative transcripts, a total of 15 *SPL* members were determined in the *C. clementina* genome (Figure 1). To date, only one mature *miR156* sequence from *Citrus* has been experimentally validated (Xu et al., 2010). Of the 15 putative *Citrus SPL* transcripts, 10 contained sequences complementary to the 20-nucleotide mature *miR156* sequence, with one or two mismatches at the 1st, 7th, or 9th nucleotide (Figure 1). Analysis of the other five *SPL* transcripts resulted in no significant matches. Alignment of the full-length protein sequences of *Arabidopsis* and *Citrus* showed no consensus sequences other than the SBP domain (not shown). Therefore, only the SBP domains (Supplementary Table 3) were used for the phylogenetic analysis (Figure 2). The results of this analysis suggested that gene multiplication occurred before separation of *Citrus* and *Arabidopsis*, which are considered to be taxonomically related. Only *Ciclev10020532* contained an SBP domain that was somewhat unique to *Citrus* (Figure 2). In general, these SPLs could be classified into three subgroups. The first one (green clade) consisted of SPLs characterized by relatively short protein sequences (<200 amino acids) and a *miR156*-binding site located within the 3′UTR of the transcript. The *Arabidopsis* members of this group, AtSPL3, AtSPL4 and AtSPL5, showed the highest homology to three short *Citrus* SPL members: Ciclev10009879, Ciclev10017104 and Ciclev10016841 containing 130, 143 and 189 amino acids, respectively. The second group (blue clade) consisted of SPLs characterized by longer protein sequences (>300 amino acids) and a *miR156*-binding site located within the ORF. The seven *Citrus SPL* members within this clade were: *Ciclev10020532*, *Ciclev10031391*, *Ciclev10032171*, *Ciclev10031834*, *Ciclev10031270*, *Ciclev10019546*, and *Ciclev10011938*. The third group (black clades) consisted of SPLs which did not contain a *miR156*-binding site. The five *Citrus* SPL members within this group were: *Ciclev10021106*, *Ciclev10004227*, *Ciclev10018697*, *Ciclev10000100*, and *Ciclev10004348*.

### Molecular characterization of *miR156*-regulated *CiSPL5*

**Figure 3:**
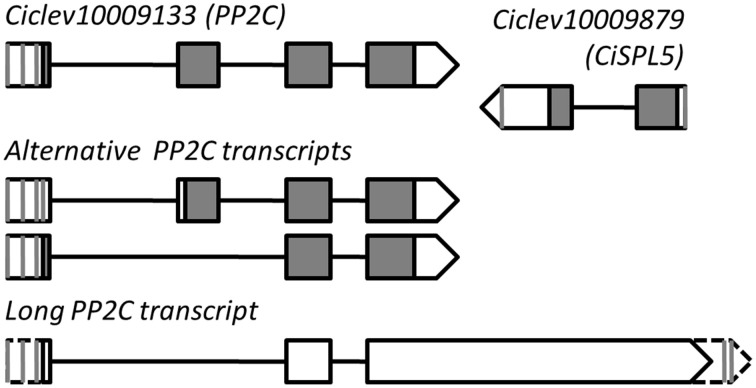
**Genomic structure of** ***CiSPL5*** **(*****Ciclev10009879*****) and its long antisense transcript**. Vertical gray lines and triangle tips indicate transcription start site or polyadenylation site identified by 5′ or 3′ RACE. Exon–intron organization of *Ciclev10009133* (*PP2C*) primary and alternative transcripts is based on the *Citrus clementina* genome database (http://www.phytozome.net/).

The roles of *SPL*s and *miR156* as regulators of phase transitions have been extensively studied in *Arabidopsis* and other annual plant species; however, considerably less research has been done with trees. To gain insight into the roles of *SPL*s in *Citrus* and understand whether the presence of a *miR156*-binding site in an *SPL* transcript constitutes a real regulatory element, we performed a molecular and functional characterization of one *Citrus SPL*, *Ciclev10009879*, whose expression had been previously studied in relation to fruit-load effect on flowering induction of *Citrus* (Shalom et al., 2012, 2014). Full-length sequencing of *Ciclev10009879* mRNA revealed that it is 843 bp long (with one alternative polyadenylation site at 750 bp) with a putative ORF encoding a 130-amino acid protein and a *miR156*-binding site located in the 3′UTR. An antisense transcript (AST) of about 2400 bp which encompasses the entire region of *Ciclev10009879* was also identified (Figure 3). Surprisingly, RACE analyses indicated that the transcription start site of this AST is located in a neighboring upstream gene, *Ciclev10009133*, encoding a putative PROTEIN PHOSPHATASE 2C (PP2C). In fact, the full-length structure of the AST was similar to one of the predicted alternative transcripts of *Ciclev10009133*, only with a longer than predicted 3′ tail (long PP2C transcript, Figure 3). The RACE analyses identified four transcription start sites and four polyadenylation sites in the AST, suggesting a complex mode of transcription. In *Citrus*, fruit set takes place during May, whereas September-October are regarded as the last time points at which fruit removal during the ON-year reverses the AB trend (Martinez-Fuentes et al., 2010). The floral induction period starts in mid-November and lasts until approximately mid-January (Davenport, 1990). Expression analysis of the long AST in buds showed that it was expressed at higher levels from May to September and lower levels from November to January (Supplementary Figure 1). However, no significant differences were detected between buds of ON- and OFF-Crop trees, and no alterations were detected following fruit removal, putting its role in flowering control by fruit load into question. As mentioned above, *Ciclev10009133* encoded a protein that is highly homologous to *Arabidopsis* PP2C (*At3g15260*; 79% identity). In the *Arabidopsis* genome, this gene is located jointly and in antisense orientation to *AtSPL5*, which belongs to the small SPLs subgroup (Figure 2). Therefore, based on SBP-domain sequence homology, protein length, *miR156*-binding site position and genomic coupling with PP2C, we determined that *Citrus SPL Ciclev10009879* is the ortholog of *Arabidopsis AtSPL5*, and it is henceforth referred to as *CiSPL5*.

### *CiSPL5* promotes flowering in arabidopsis and is repressed by *miR156*

**Figure 4:**
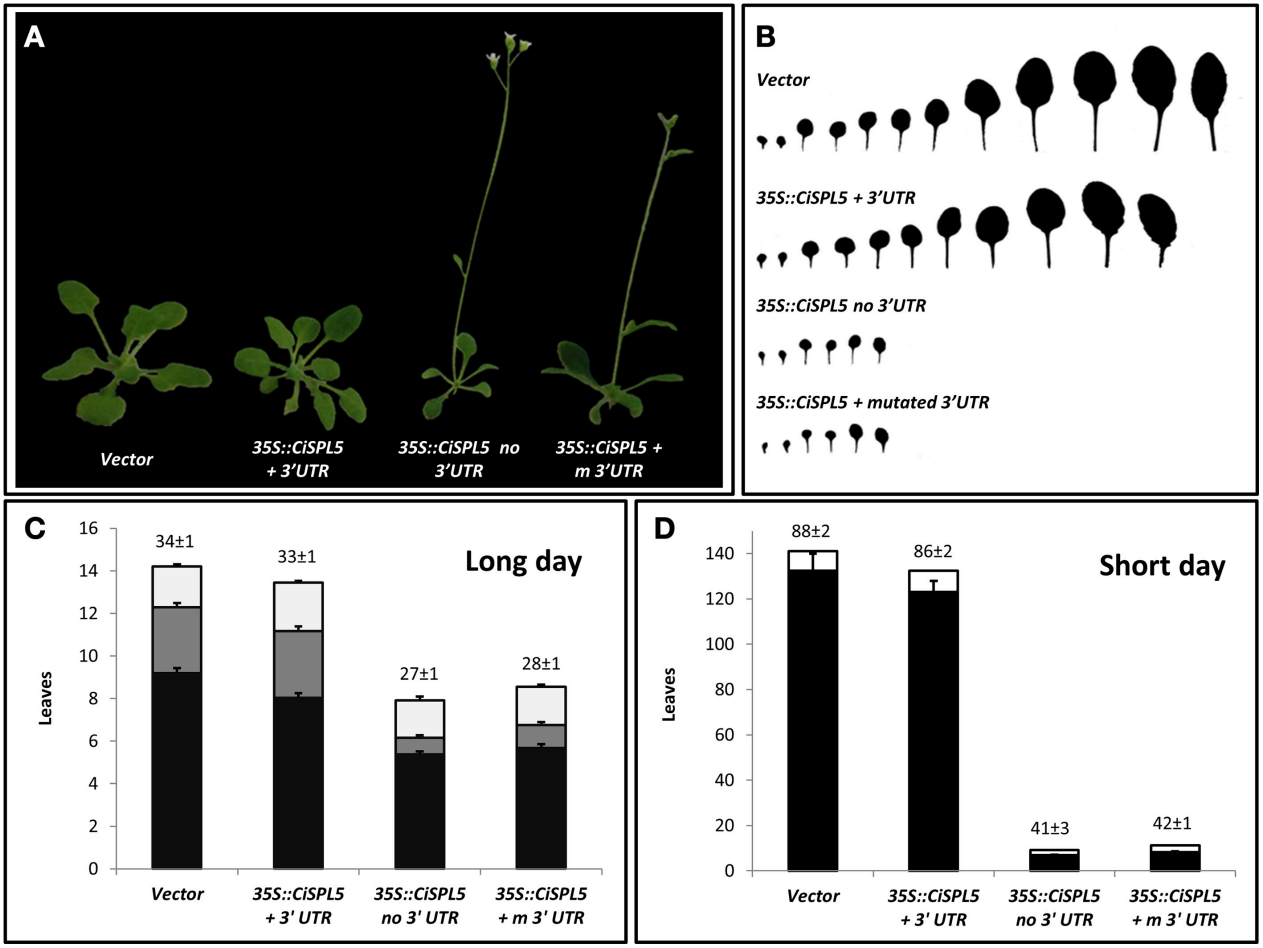
**CiSPL5 promotes flowering in** ***Arabidopsis*** **and is repressed by** ***miR156***. **(A)** Morphology of transgenic plants expressing *miR156*-sensitive (*35S::CiSPL5 + 3′UTR*) and *miR156*-insensitive (*35S::CiSPL5 no3′UTR*, *35S::CiSPL5 + m3′UTR*) versions of *CiSPL5* under the regulation of the 35S promoter. (**B**) Morphology of rosette leaves in the transgenic plants. (**C**) The number of leaves without abaxial trichomes (black bars), with abaxial trichomes (gray bars), cauline leaves (white bars) and flowering time (days after planting, top of bar ± SE) for the transgenic lines under long days. (**D**) The number of rosette leaves (black bars), cauline leaves (white bars) and flowering time (days after planting, top of bar ± SE) for the transgenic lines under short days.

Transgenic *Arabidopsis* plants expressing *CiSPL5* under the control of CaMV 35S promoter in either *miR156*-sensitive (containing the native *miR156*-binding site) or *miR156*-insensitive forms (not containing the native *miR156*-binding site) were generated and phenotyped. Three constructs were used: *CiSPL5* ORF transcript with its normal 3′UTR (*miR156*-sensitive, *35S::CiSPL5* + 3'*UTR*, Supplementary Figure 2-1), *CiSPL5* ORF transcript lacking the 3′UTR (*miR156*-insensitive, *35S::CiSPL5 no3′UTR*, Supplementary Figure 2-2) and *CiSPL5* ORF transcript with 10 mutations in the 3′UTR *miR156*-binding site (*miR156*-insensitive, *35S::CiSPL5 + m3′UTR*, Supplementary Figure 2-3). Characterization of the *CiSPL5*-transgenic lines indicated similarity between the *35S::CiSPL5* + 3'*UTR* and control (Figure 4A), producing normal size rosette leaves (Figure 4B). The former flowered after ~33 days under long days (Figure 4C) or ~86 days under short days (Figure 4D), which was slightly ahead of the control (34 days and 88 days, respectively). In contrast, both *35S::CiSPL5 no3′UTR* and *35S::CiSPL5 + m3′UTR* lines produced much smaller and fewer rosette leaves before flowering occurred (Figures 4A,B). Furthermore, time to flowering in these lines was significantly reduced under long days and even more dramatically under short days (Figures 4C,D, respectively). *35S::CiSPL5 no3′UTR* and *35S::CiSPL5 + m3′UTR* lines had a significantly reduced number of rosette leaves with abaxial trichomes (Figure 4C), characteristic of the adult phase (Telfer et al., 1997), compared to *35S::CiSPL5 + 3′UTR* lines and the control lines. Moreover, the formation of abaxial trichomes in *35S::CiSPL5 no3′UTR* and *35S::CiSPL5 + m3′UTR* lines initiated earlier than in *35S::CiSPL5 + 3′UTR*.

### Seasonal expression patterns of some *CiSPLs* and patterns following fruit removal coincide with flowering induction

**Figure 5:**
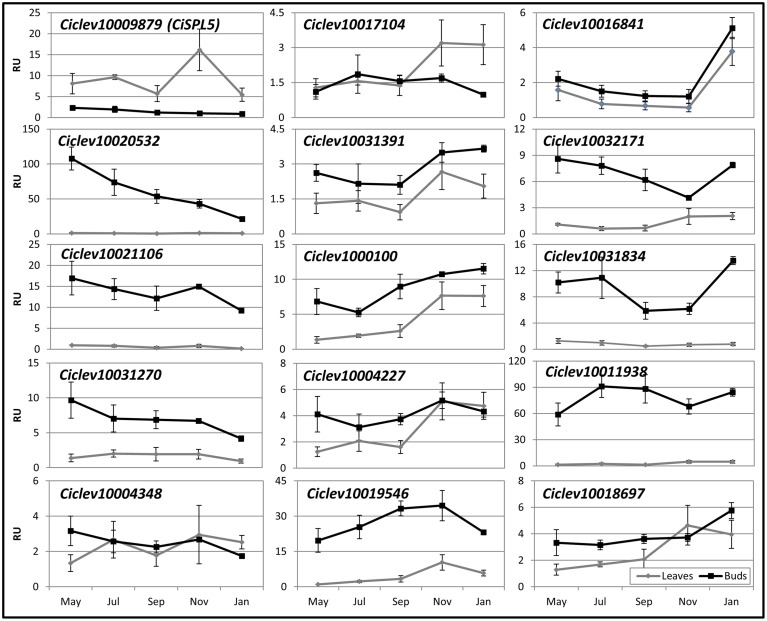
**Seasonal expression patterns of** ***Citrus SPL*****s in OFF-Crop leaves and buds.** The mRNA levels (RU, relative units) of the indicated genes were determined in OFF-Crop leaves (gray lines) and buds (black lines) at the indicated months. The numbers are mean values of three independent biological replicates ± SE. The pattern in the bud is shown in Supplementary Figure 3 with extended axis.

As woody perennials, *Citrus* trees undergo a series of developmental changes deriving from seasonal environmental and endogenous cues. Some of these changes, such as the shift to flowering and/or vegetation, might be regulated, at least in part, by SPLs. Therefore, we studied their expression patterns in leaves and buds of OFF-Crop trees, expected to flower the following spring, before and during the flowering induction period from November to January. Nine out of fifteen *SPL*s (*Ciclev10020532*, *Ciclev10021106*, *Ciclev10031270*, *Ciclev10031391*, *Ciclev1000100*, *Ciclev10019546*, *Ciclev10032171*, *Ciclev10031834*, and *Ciclev10011938*) were expressed at higher levels in buds than in leaves at most time points (Figure 5), whereas 4 out of 15 *SPLs* showed similar expression levels in buds and leaves at most time points. The mRNA levels of one *SPL*, *Ciclev10017104*, were similar in leaves and buds from May until September, but were higher in leaves from November to January. However, the mRNA of *CiSPL5* was exceptional in that at all tested time points, it showed significantly higher levels in leaves than in buds. While *CiSPL5* expression in leaves was relatively stable, with a transient threefold increase in November, its expression in buds gradually decreased from May to January (Figure 5, Supplementary Figure 3), consistent with our previous report (Shalom et al., 2012). Similarly, the mRNA levels of *Ciclev10020532*, *Ciclev1002110*6, and *Ciclev10031270* decreased moderately throughout the season in the buds. In contrast, the mRNA levels of *Ciclev10019546* in buds showed a moderate increase from May to November followed by a slight decrease in January, similar to the trend in leaves. Another *SPL* whose expression is worth mentioning is *Ciclev10016841*, which showed a sharp increase in mRNA levels from November to January.

**Figure 6:**
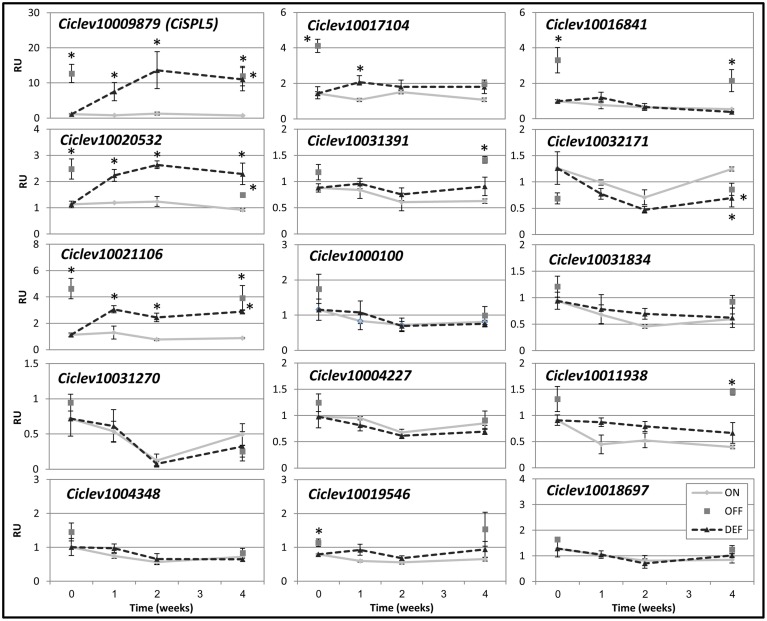
**Expression patterns of** ***Citrus SPL*****s in buds following fruit removal.** The mRNA levels (RU, relative units) of the indicated genes were determined in ON-Crop (ON, diamonds), OFF-Crop (OFF, squares), and de-fruited (DEF, dashed lines) trees at the indicated weeks after de-fruiting. The numbers are mean values of three independent biological replicates ± SE. Asterisks represent significant difference (*P* = 0.05) from ON-Crops at the same time point. *CiSPL5* graph was presented in Shalom et al. (2014).

As heavy fruit load inhibits flowering induction, the mRNA levels of *SPLs* were also investigated in buds of ON- and OFF-Crop trees and following fruit removal (de-fruiting). Most of the *SPL*s were of similar levels in ON- and OFF-Crop buds, and were not altered significantly by de-fruiting (Figure 6). However, the mRNA levels of *Ciclev10020532* and *Ciclev10021106* were higher in OFF-Crop vs. ON-Crop buds, and they were significantly up-regulated (two- to threefold) 1 week after de-fruiting. In addition, the level of *Ciclev10016841* mRNA was significantly higher (by about threefold) in OFF-Crop buds than in ON-Crop buds; however, its level was not altered by de-fruiting.

### Fruit removal alters the expression of SPL-related miRNA genes, but not necessarily abundance of the mature miRNA

**Figure 7:**
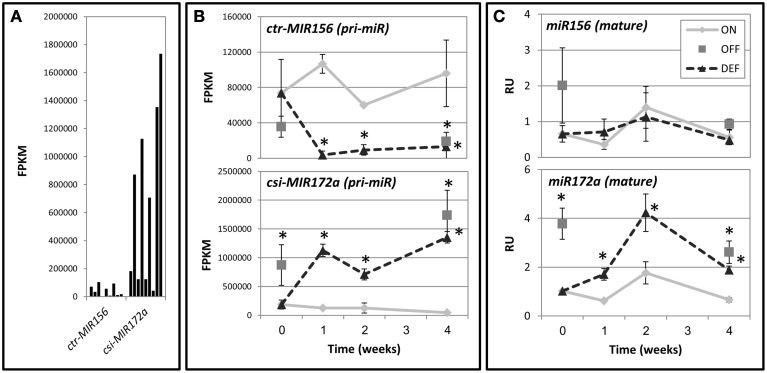
***miR156*** **and** ***miR172*** **levels in buds following fruit removal.** (**A**) Expression levels of *ctr-MIR156* and *csi-MIR172a* genes are based on transcriptomic data and are represented by FPKM-values, which normalize the read count by the length of the fragment and the total number of mapped reads. (**B**) Expression patterns of *ctr-MIR156* and *csi-MIR172a* genes after fruit removal. (**C**) Abundance of the mature *miR156* and *miR172a* sequences after fruit removal. For **(B,C)**, values were determined in ON-Crop (ON), OFF-Crop (OFF), and de-fruited (DEF) trees at the indicated weeks after de-fruiting. The numbers are mean values of three independent biological replicates ± SE. Asterisks represent significant difference (*P* ≤ 0.05) from ON at the same time point. pri-miR, primary mRNA.

Primary miRNAs (pri-miRs) are capped and polyadenylated non-coding RNA transcripts containing mature miRNA sequences (Voinnet, 2009). Cleavage of the primary miRNA transcript eventually results in the release of biologically active mature miRNA and ultimately, the degradation or translational repression of mRNA targets. The miRBase database (http://www.mirbase.org/) contains sequence data (precursor and mature) for 75 miRNAs from *Citrus*. We recently conducted an RNA deep-sequencing analysis of *Citrus* buds following fruit removal (Shalom et al., 2014). In the current work, we aligned the RNA deep-sequencing data to the *Citrus* precursor sequences from miRBase. Results of this analysis indicated that about two-thirds of them are expressed in buds; however, only a few were significantly affected by fruit load. Among these were *ctr-MIR156* and *csi-MIR172a*, with the latter expressed to much higher levels (Figure 7A). Expression levels of *ctr-MIR156* were significantly higher in ON-Crop buds as compared to OFF-Crop buds and decreased following de-fruiting (Figure 7B). In contrast, expression levels of *csi-MIR172a* showed the opposite trend, with OFF-Crop buds and buds after de-fruiting showing higher expression levels than ON-Crop buds. Alterations in both genes occurred as early as 1 week after de-fruiting (Figure 7B). Abundance analysis of the mature miRNA sequences was performed next. While *miR156* abundance was not affected by fruit presence, *miR172* was more abundant in OFF-Crop buds than ON-Crop buds, and increased significantly (two to threefold) after de-fruiting (Figure 7C).

## Discussion

### *SPL* gene family in *Citrus*

Genes containing the SBP domain were originally identified in *Antirrhinum majus* and were named SQUAMOSA PROMOTER BINDING PROTEIN based on their ability to interact with the promoter of the floral meristem identity gene *SQUAMOSA* (Klein et al., 1996). Since then, SPLs have been identified and classified in a number of plant species, including *Arabidopsis* (Rhoades et al., 2002), rice (Xie et al., 2006), tomato (Salinas et al., 2012), and grape (Hou et al., 2013). Similar to other plants, about two-thirds of the *Citrus SPL*s contain sequences complementary to *miR156*. In *Arabidopsis*, 3 of the 10 *miR156*-regulated SPLs, AtSPL3/4/5, differ from the others by the position of their *miR156*-binding site—it is located in the 3′UTR and is believed to have moved there *via* exon degeneration (Guo et al., 2008)—and by their relatively short protein size, which mainly comprises the SBP domain (Wu and Poethig, 2006; Yamaguchi et al., 2009). Overexpression of each of these SPLs shortens the juvenile period and induces early flowering (Wu and Poethig, 2006). AtSPL3 activates *LEAFY (LFY), FRUITFUL (FUL)*, and *APETALA1 (AP1)* by directly binding their promoter regions and evidence suggests that AtSPL4 and AtSPL5 have overlapping functions (Yamaguchi et al., 2009). Our phylogenetic analysis showed that AtSPL3/4/5 have three close orthologs in *Citrus*, with similar structural characteristics and therefore potentially similar functions. By its structure and genomic localization (i.e., proximity to a *PP2C*-like gene), *Ciclev10009879* (*CiSPL5*) is suggested to be the ortholog of *Arabidopsis AtSPL5*.

### Antisense transcription within *CiSPL5*

We identified an AST which encompasses the *CiSPL5* genomic region. A large number of overlapping transcripts in antisense orientation with the potential to form double-stranded RNA structures have been identified in the *Arabidopsis* genome (Jen et al., 2005; Wang et al., 2005), for instance, in *AtSPL3* (Wu and Poethig, 2006). Although the role of these ASTs remains unclear, they might function as an additional level of regulation. The identified AST is a long variant of a neighboring gene encoding PP2C-like protein homologs of *At3g15260*. Some PP2C proteins play a role in abscisic acid (ABA) signaling (Umezawa et al., 2009). ABA homeostasis is affected by fruit load (Shalom et al., 2014), and the decrease in the AST's expression during the winter months somewhat resembles the expression pattern of *CiSPL5* (although it does not respond to fruit load). Therefore, exploration of ABA's possible signaling role in *CiSPL5* regulation is warranted.

### Do *SPL*s and *miR172* play a role in fruit-load effect on flowering in *Citrus*?

*CiSPL5* was able to accelerate *Arabidopsis* flowering while *miR156* repressed its action (Figure 4). Although no direct evidence was provided, it was likely that *CiSPL5* regulated phase transition in *Citrus*, as well. A significant difference in its expression was detected between ON- and OFF-Crop buds, and it was induced by de-fruiting (Shalom et al., 2012, 2014), raising the posebility it also played a role in AB regulation. However, the findings that the levels of *miR156* in *Citrus* buds were not affected by fruit load (Shalom et al., 2012, Figure 7C), questioned the possibility that fruit load regulated *CiSPL5* expression through *miR156*. Previous investigations in various plant species demonstrated that *miR156* was predominant in juvenile tissues, whereas *miR172* was induced in adult tissues (Aukerman and Sakai, 2003; Chen, 2004; Lauter et al., 2005; Wu and Poethig, 2006; Chuck et al., 2007). Our transcriptomic data (Figure 7) showed low expression levels of *ctr-MIR156* relative to *csi-MIR172a*, which is in agreement with the accepted dogma of their respective roles in juvenile and adult plants. This could explain why the levels of *miR156* did not correlate with fruit load. Additional finding was the lack of correlation between the expression patterns of *ctr-MIR156* and mature *miR156* (Figure 7). It might be that the final level of this miRNA was ultimately determined by a combination of factors, including additional active *miR156* genes or small interfering (si) RNAs.

Unlike *miR156*, *miR172a* increased both at the precursor and mature levels following de-fruiting (Figure 7). In *Arabidopsis*, *miR172* transcription is induced by AtSPL9 and AtSPL10 (Wu et al., 2009), and in turn promotes flowering by repressing *AP2*-like TFs which negatively regulate *FT* expression (Mathieu et al., 2009). Could this also be the case in *Citrus* fruit load-affected flowering? In parallel to *csi-MIR172a* induction, three *CiSPL* genes were up-regulated following de-fruiting in the bud (Figure 6): *CiSPL5* (*Ciclev10009879*), *Ciclev10020532*, and *Ciclev10021106*. While *Ciclev10020532* has no close ortholog in *Arabidopsis* (Figure 2), *Ciclev10021106* is the ortholog of *AtSPL8*, found to play important roles in the regulation of male fertility (Xing et al., 2010) and gynoecium patterning (Xing et al., 2013). Whether *Ciclev10021106* has similar roles in *Citrus* is unclear; however, it is not targeted by *miR156*. Taken together, it is tempting to speculate that some of these SPLs regulate *miR172a* expression by a mechanism similar to that in *Arabidopsis*, as mentioned above.

### *miR156*'s negative regulation of *CiSPL5* in *Arabidopsis* can be bypassed through the photoperiod pathway

In addition to flowering promotion, *CiSPL5* shortened the juvenile phase, as reflected by positive regulation of abaxial trichome production, which is characteristic of adult leaves. This has also been shown for other SPLs (Cardon et al., 1997; Wang et al., 2009; Wu et al., 2009). While long day promotes *Arabidopsis* flowering, under short day, flowering occurs after a long period of vegetative growth, as a result of *miR156* down-regulation accompanied by a gradual increase in *AtSPL3* and *AtSPL9* expression (Wang et al., 2009). Shifting plants from short days to long days leads to a rapid increase in *AtSPL3* and *AtSPL9* expression while *miR156* levels remain unchanged. Our functional analysis of *CiSPL5* in *Arabidopsis* supports the notion that the negative regulation by *miR156* can be bypassed through the photoperiod pathway; when overexpressed in *miR156*-insensitive forms, CiSPL5 accelerated flowering regardless of day length. The normal phenotype of lines overexpressing *CiSPL5* in a *miR156*-sensitive form indicated that flowering in these plants is affected by endogenous factors, such as an age-dependent pathway (short day) or the photoperiod pathway (long day).

### Conflict of interest statement

The authors declare that the research was conducted in the absence of any commercial or financial relationships that could be construed as a potential conflict of interest.

## Supplementary material

Supplementary Figure 1 **Expression of *CiSPL5* antisense transcript (long *PP2C* transcript) in buds**. The mRNA levels (RU, relative units) of the long *PP2C* transcript were determined in ON-Crop (ON) and OFF-Crop (OFF) buds at the indicated months (upper graph) and in ON-Crop (ON) and de-fruited (DEF) trees at the indicated weeks after de-fruiting (lower graph) by nCounter analysis (described in Shalom et al., 2014). The numbers are mean values of three independent biological replicates ± SE.

Supplementary Figure 2 **Constructs used for constitutive expression of *CiSPL5* with different modifications**. The sequences of the native or mutated *miR156* target sites are illustrated. The native site is indicated by a dotted black line and a mutated site is indicated by a dotted gray line.

Supplementary Figure 3 **Seasonal expression pattern of *CiSPL5* in OFF-Crop buds**. The mRNA levels (RU, relative units) of *CiSPL5* were determined in OFF-Crop buds at the indicated months. The numbers are mean values of three independent biological replicates ± SE.

Supplementary Table 1 **List of primers used in this study**.

Supplementary Table 2 **Precursor sequences of *ctr-MIR156* and *csi-MIR172a* from miRBase (http://www.mirbase.org/)**.

Supplementary Table 3 **SBP-domain sequences of all 15 SPLs from *Citrus***.

Supplementary Table 4 **RACE primers specific for *Citrus* SPL**.
