# Supplementary figures and images for: Molecular characterization of SQUAMOSA PROMOTER BINDING PROTEIN-LIKE (SPL) gene family from Citrus and the effect of fruit load on their expression
Source: Front Plant Sci. 2015 May 27;6:389. doi: 10.3389/fpls.2015.00389 (PMC4443640; doi:10.3389/fpls.2015.00389)

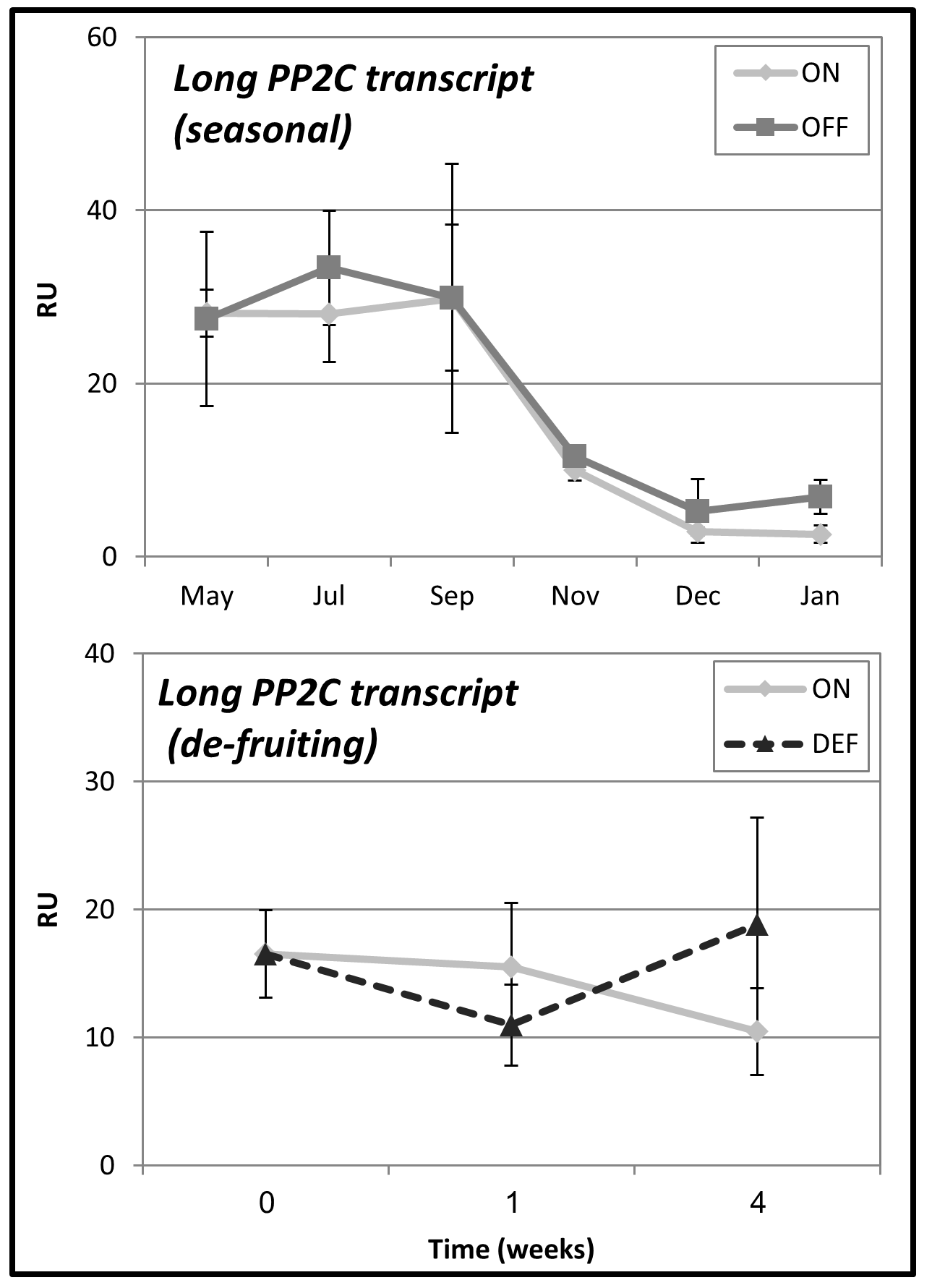

Supplement: Supplementary Figure 1 — Expression of CiSPL5 antisense transcript (long PP2C transcript) in buds. The mRNA levels (RU, relative units) of the long PP2C transcript were determined in ON-Crop (ON) and OFF-Crop (OFF) buds at the indicated months (upper graph) and in ON-Crop (ON) and de-fruited (DEF) trees at the indicated weeks after de-fruiting (lower graph) by nCounter analysis (described in Shalom et al., 2014). The numbers are mean values of three independent biological replicates ± SE. [file Image1.TIF]

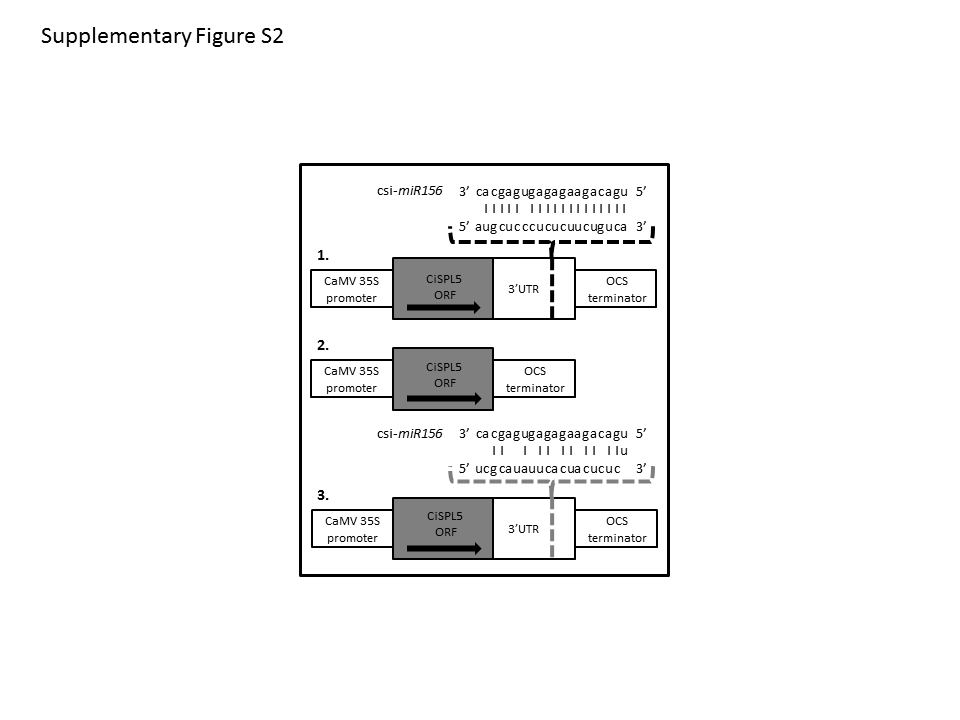

Supplement: Supplementary Figure 2 — Constructs used for constitutive expression of CiSPL5 with different modifications. The sequences of the native or mutated miR156 target sites are illustrated. The native site is indicated by a dotted black line and a mutated site is indicated by a dotted gray line. [file Image2.TIF]

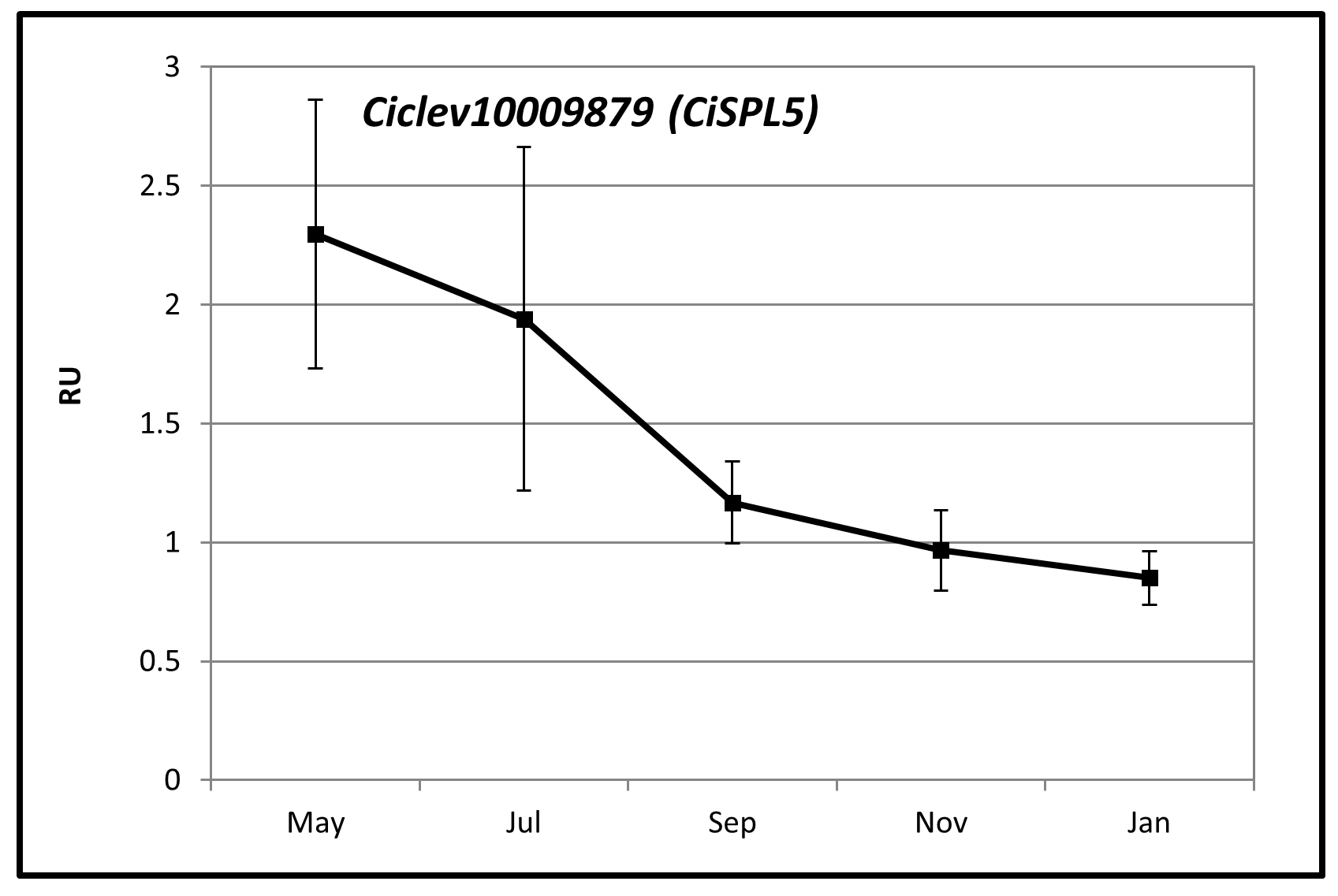

Supplement: Supplementary Figure 3 — Seasonal expression pattern of CiSPL5 in OFF-Crop buds. The mRNA levels (RU, relative units) of CiSPL5 were determined in OFF-Crop buds at the indicated months. The numbers are mean values of three independent biological replicates ± SE. [file Image3.TIF]
